# Soybean Cyst Nematodes Influence Aboveground Plant Volatile Signals Prior to Symptom Development

**DOI:** 10.3389/fpls.2021.749014

**Published:** 2021-09-29

**Authors:** Nasie Constantino, Yeonyee Oh, Erdem Şennik, Brian Andersen, Michael Warden, Ömer Oralkan, Ralph A. Dean

**Affiliations:** ^1^Department of Entomology and Plant Pathology, North Carolina State University, Raleigh, NC, United States; ^2^Electrical and Computer Engineering, North Carolina State University, Raleigh, NC, United States; ^3^Department of Nuclear Engineering, North Carolina State University, Raleigh, NC, United States; ^4^BASF Plant Science, Research Triangle, NC, United States

**Keywords:** soybean cyst nematode, VOCs, early disease detection, GC-MS, soybean

## Abstract

Soybean cyst nematode (SCN), *Heterodera glycines*, is one of the most destructive soybean pests worldwide. Unlike many diseases, SCN doesn't show above ground evidence of disease until several weeks after infestation. Knowledge of Volatile Organic Compounds (VOCs) related to pests and pathogens of foliar tissue is extensive, however, information related to above ground VOCs in response to root damage is lacking. In temporal studies, gas chromatography-mass spectrometry analysis of VOCs from the foliar tissues of SCN infested plants yielded 107 VOCs, referred to as Common Plant Volatiles (CPVs), 33 with confirmed identities. Plants showed no significant stunting until 10 days after infestation. Total CPVs increased over time and were significantly higher from SCN infested plants compared to mock infested plants post 7 days after infestation (DAI). Hierarchical clustering analysis of expression ratios (SCN: Mock) across all time points revealed 5 groups, with the largest group containing VOCs elevated in response to SCN infestation. Linear projection of Principal Component Analysis clearly separated SCN infested from mock infested plants at time points 5, 7, 10 and 14 DAI. Elevated Styrene (CPV11), D-Limonene (CPV32), Tetradecane (CPV65), 2,6-Di-T-butyl-4-methylene-2,5-cyclohexadiene-1-one (CPV74), Butylated Hydroxytoluene (CPV76) and suppressed Ethylhexyl benzoate (CPV87) levels, were associated with SCN infestation prior to stunting. Our findings demonstrate that SCN infestation elevates the release of certain VOCs from foliage and that some are evident prior to symptom development. VOCs associated with SCN infestations prior to symptom development may be valuable for innovative diagnostic approaches.

## Introduction

As one of the major nutritional foods in human diets that provides high quality proteins and oil, soybeans are one of the most economically important agricultural crops globally (Whitham et al., 2016; Liu et al., 2020). Considering its value and the increasing world population, soybean production is in high demand to aid global food security. Unfortunately, production levels can be dramatically decreased by various abiotic and biotic stresses, one of the most prominent being *Heterodera glycines* (Niblack et al., 2006). *H. glycines* commonly referred to as the soybean cyst nematode (SCN) is responsible for causing upwards of $1.2 billion of yield loss each year in the USA (Davis et al., 2004). The nematode penetrates soybean roots as second-stage juveniles (J2s) where they grow and modify the plant root tissue leaving swollen lemon-shaped females exposed on the root surface. Eggs are laid turning her body into a protective brown cyst. The extensive root damage leads to the aboveground symptoms of plant stunting and yellowing. However, these aboveground indicators are difficult to visualize until late infestation and there has been extensive root damage leading to significant yield loss (Niblack et al., 2006). Traditionally, SCN has been managed through a combination of nematicides, SCN-resistant soybean varieties, and crop rotation (Niblack, 2005). However, some of these current practices are losing their effectiveness or being phased out. Through genetic variability, a short life cycle, and numerous progeny SCN populations have become resilient against nematicides and resistant soybean varieties (Davis et al., 2008; Kikuchi et al., 2017). Therefore, it is imperative that new management approaches be developed to tackle this pest. The detection of SCN prior to symptom development would likely be of practical value.

Plants produce a myriad of volatile organic compounds (VOCs) and several thousand have been identified to date (Baldwin, 2010). In general plant volatiles are produced from several metabolic pathways, including the terpene, phenylpropanoids and benzenoids, fatty acid derivatives, and amino acid derivatives, along with several species-specific compounds that are not included in the major classes (Dudareva et al., 2013). It is well known that plant tissues release specific blends of VOCs in response to abiotic and biotic stress (Niinemets et al., 2013; Vivaldo et al., 2017). VOCs have been well studied to be plant signals that mediate intra- and interspecies communications in relation to herbivore and microbe interactions (Bitas et al., 2013). In response to insect damage plants, including soybean, release herbivore-induced plant volatiles (HIPVs) which are largely composed of terpenes, nitrogenous compounds, green leafy volatiles, and indoles (Michereff et al., 2011; Strapasson et al., 2016; Aljbory and Chen, 2018). The HIPVs induce plant defenses, attract parasitic insects, and warn adjacent plants of an impending attack (Bitas et al., 2013; Rowen and Kaplan, 2016; Aartsma et al., 2017). During microbial infections, plants have been shown to release elevated levels of volatile aromatics, terpenes, fatty acid derivatives, and nitrogen-containing compounds, along with the volatile plant hormones, methyl jasmonate, and methyl salicylate (Hammerbacher et al., 2019). Depending on the combination of plant and pathogen, emitted VOCs can induce resistance or susceptibility, along with attracting or repelling various insects (Hammerbacher et al., 2019). The composition of these VOCs is often specific and can be diagnostic of the type of stress or pathogen (Sharifi and Ryu, 2018). To date, only a few studies of root pathogens, including nematodes, affecting the foliar VOC emissions have been reported (Bezemer and Dam, 2005; Hong et al., 2010; Lin et al., 2017; Castorina et al., 2020). Nevertheless, given the evidence that root stress influences foliar VOCs, we hypothesize that infestation of soybean roots with SCN results in the release of VOCs from foliar tissue potentially before the appearance of obvious symptoms. The detection of these VOCs may provide a novel means of early disease detection. In this study, we employ GC-MS analysis to identify and examine the temporal profile of VOCs released by foliar tissues following infestation of roots with SCN. We show that the VOC profile changes during infestation, and that several VOCs can be detected during early time points, prior to symptom development. In addition to providing knowledge of specific VOCs produced by soybean during SCN infestation, the identification of these VOC biomarkers will help facilitate the development of e-nose technology for the early detection of SCN. This research also further advances knowledge on below- and aboveground responses to pests and pathogens.

## Materials and Methods

### Plant Material

The soybean, *Glycine max* variety Roy provided by BASF Seed, Soil, Systemicity Advanced Research Laboratory APR/IA RTP NC USA was used in this study. Seeds were planted at a depth of 1.5-cm in SC10L Super Cell “Cone-tainers” (3.8-cm dia, 21-cm tall, 164 cm^3^, Hummert International) containing two cotton balls at the bottom to prevent substrate loss. The substrate was a mixture of dry Patio sand and sandhills loamy sand soil (Sands and Soils, Durham, NC) to achieve a final ratio of 3:1. Sandhills loamy sand soil was first sifted with a No. 10 sieve and then incorporated by hand with dry Patio sand. Plants were grown in the greenhouse maintained at 24+/−1°C with 14 h of light per day under automatic watering (4 min, 4 times a day) until the first trifoliate began to emerge (~2 weeks after planting). After infestation at 2 weeks of growth, watering switched to 8 min, 3 times a day and was later switched to 10 min, 3x a day 2 weeks after infestation.

### Nematode Infestation

*H. glycines*, Race 2, cysts were harvested from colony infested soybean roots and ground in sieves to release the eggs. The eggs were then collected in another sieve and washed with distilled water into a beaker. Next the egg solution was placed on a moist coffee filter suspended over distilled water. The juveniles (J2s) were subsequently allowed to hatch over several (3–5) days and swim through the coffee filters into the water below. Two-week-old soybean plants were infested with 10,000 J2s in 10 ml distilled water. The 10 ml of J2 water solution was distributed equally between two 2 cm deep holes on either side of the soybean seedling. Mock infestation was conducted using distilled water. At the end of each experiment [~33 days after infestation (DAI)] roots were rinsed, and cysts counted to confirm infection with mean cysts numbers of 1,846 and 1,059 for the first and second experiments, respectively. No cysts were observed on mock treated plants.

### Experimental Setup for VOC Collection

**Figure 1:**
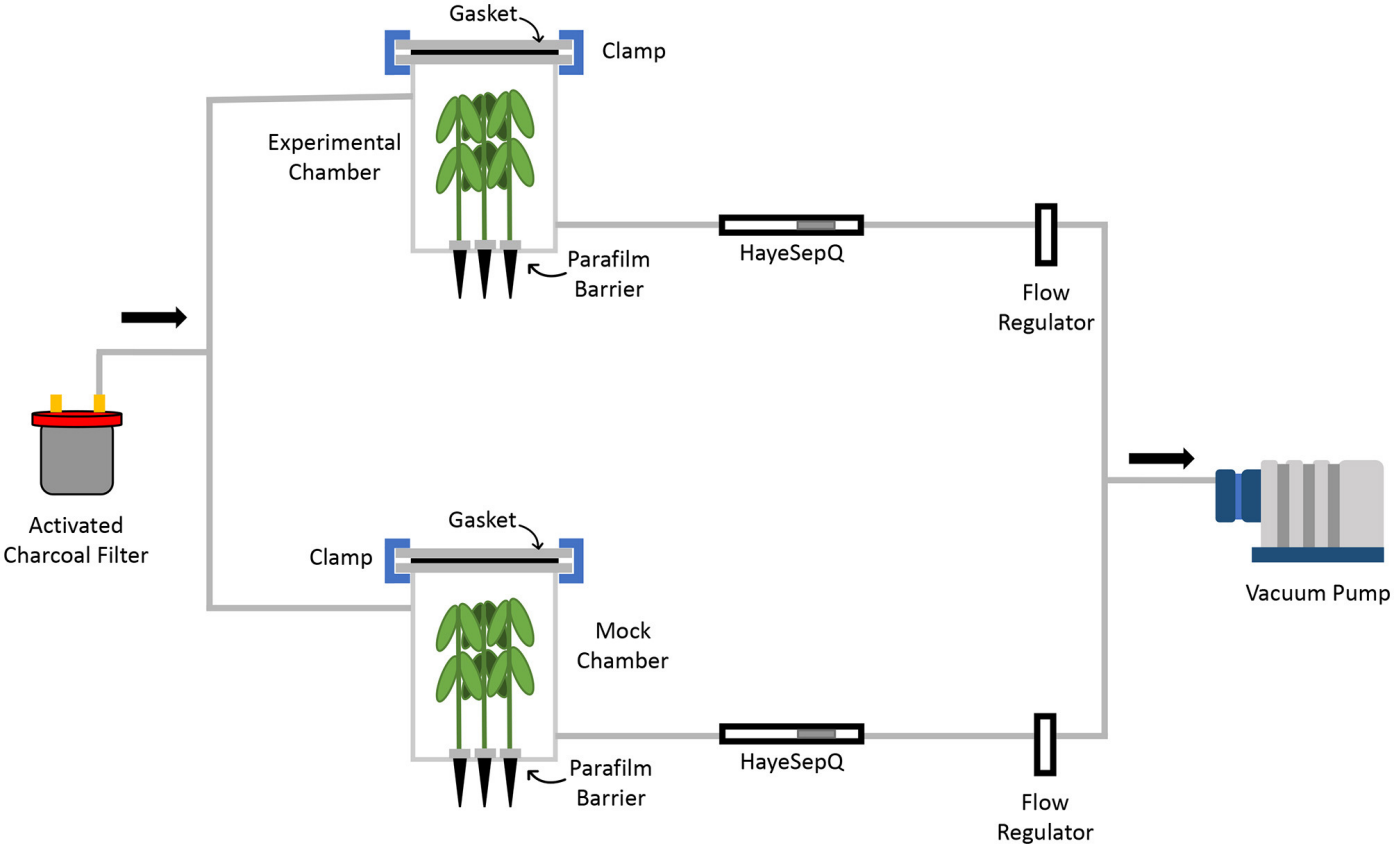
Schematic diagram of volatile collection system for GC-MS analysis. Air entering the chamber was scrubbed using activated charcoal. Volatiles were pulled from the chambers *via* a vacuum pump and collected by HayeSepQ resin for GC-MS analysis. To ensure only foliar volatiles were collected a parafilm barrier was placed at the base of each soybean plant.

A custom fabricated air flow volatile collection system was designed specifically for this research (Figure 1). Both the chamber and chamber lid were constructed with polyethylene terephthalate glycol (PETG) plastic (ePlastics; San Diego, CA 92123). At the bottom of each chamber 4 holes in a square pattern were drilled to allow cone-trainers to be inserted, allowing for the foliar part of the plants to be in the chamber (Supplementary Figure 1). The chamber contained single inlet and outlet ports located 4 in from the top and bottom of the chamber, respectively. To ensure an airtight seal for air flow within the system a PETG gasket was placed between the lid and the chamber secured using clamps. All 3/8 in ID tubing used for the system consisted of polyethylene terephthalate (PET) plastic (United States Plastic Corp., Lima, Ohio, USA). To ensure all volatiles collected were from the headspace of the treated plants, all air was scrubbed with activated charcoal before entering the system. Air was vacuumed through the filter into the chambers where the headspace was pulled across 35 mg of HayeSep® Q 80/100 absorption resin (Analytical Research Systems, Inc., Gainesville, FL, USA) packed tightly into Supelco glass tube (6 mm × 4 mm × 7; Supelco, Bellefonte, PA). All air was pumped out of the system using a vacuum pump and air flow was adjusted to 500 ml/min.

### Volatile Headspace Collection

Volatiles were collected from both mock treated and SCN infested plants. Each treatment consisted of 5 replicates with 4 plants per replicate. Time points for this study were day 1, 3, 5, 7, 10, and 14 DAI. The experiment was repeated once. The lower stem of each plant was wrapped with Parafilm to separate the soil and roots from the above ground tissue, to ensure the collection of only foliar volatiles (Figure 1). Once plants were placed and sealed into the chambers, volatiles flowed across the HayeSepQ collection resin in the Supelco glass tube for 1 hr. Immediately after collection, volatiles were eluted from the resin by adding 300-μl Dichloromethane with 5 nmol/L n-Octane as an internal standard to the glass tube. Each eluent (~100 μl) was sealed into 2 ml vials with 0.25 ml inserts and stored at −80°C (Item numbers 89235-502, 10058-622, 10059-168 VWR International Inc.).

### GC-MS Analysis

Samples were analyzed using a gas chromatography (GC) system (7890A) paired with a mass spectrometer (5975C) (Agilent Technologies, Santa Clara, CA, USA). The GC used a non-polar HP-5MS column (30 m × 0.25 mm ID, 0.25 mm ID, 0.25 μm of film thickness; Agilent Technologies). A 2-μl sample was injected into the GC and was run on splitless mode with a carrier gas of Nitrogen. Initial temperature was 32°C with an increase of 8°C/min until a final temperature of 280°C. Temperature was increased to 325°C between samples. Hold time was 3 mins. ChemStation software (Agilent Technologies) was used for data acquisition along with the library database W9N17.L (Wiley and NIST) to initially identify the volatiles by their mass spectra. Volatiles were later confirmed by comparing their retention times and fragmentation pattern to those of standard reference compounds (Supplementary Table 1) along with the manual inspection of mass spectra (Restek Corporation, Bellefonte, PA 16823).

### GC-MS Alignment of Retention Times

**Figure 2:**
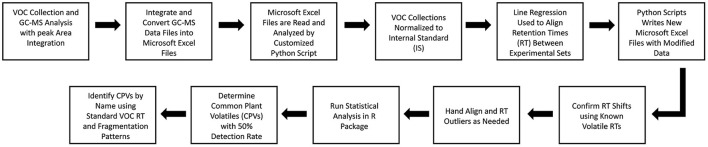
Experimental data processing and analysis pathway for the VOC peak alignment with linear regression approach.

GC-MS data files were integrated and transferred into Microsoft Excel spreadsheets. A custom Python script was then deployed to extract the retention times (min), absolute area, top hit names (1–20), and the quality of the identification based on the W9N17.L database library for each compound estimated. The script used a variable window size to organize compounds from various samples and experiments based on retention time into groups. A linear regression based on the retention time of known volatiles were optionally applied to correct for differences in the recorded retention times between GC-MS runs caused by environmental fluctuations and other factors. Once the desired retention time corrections and sorting had been performed, the sorted volatile groups were recorded and transferred into a Microsoft Excel spreadsheet. Any outliers were hand aligned and the modified data was statistically analyzed (Figure 2).

### Statistical Analysis and Data Visualization

After alignment, common plant volatiles (CPV) were identified for further analysis. To be categorized as a CPV, the VOC must be detected at every time point and in at least 50% of treatment replicates. Volatile compounds were normalized to units of n-Octane (based on area under peak). For each CPV, the ratio of areas (normalized to nmol/L Octane) for infested vs. mock was calculated for each replicate. Statistical analysis was evaluated by the randomization (permutation) test using 10,000 randomizations performed in the R package. CPVs were considered significant when *P* ≤ 0.05. Orange, an open-source data mining toolbox developed in Python with machine learning and data visualization, was used for hierarchical clustering, heatmaps, Principal Component Analysis and linear projections (Demsar et al., 2013).

## Results

### SCN Infested Soybeans Exhibit Stunting at 10 Day After Infestation (DAI)

**Figure 3:**
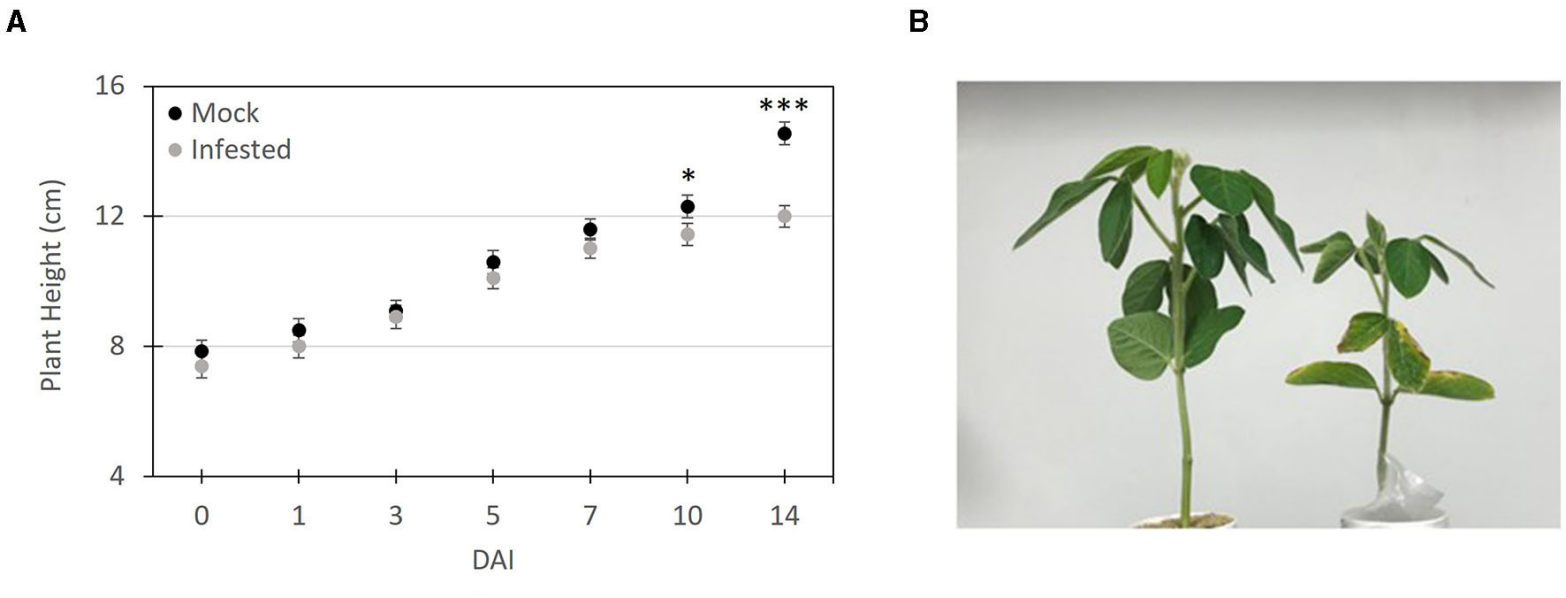
Symptom progression of soybean seedlings infested with SCN. **(A)** Height measurements of SCN infested and mock infested plants at 1, 3, 5, 7, 10 and 14 days after infestation (DAI). **(B)** Image of SCN infested plant (right) and mock infested plant (left) at 14 DAI. The plant height was measured from the soil line to the top leaf. The data shown represents an average ± SE plant height and significant differences based on Student's *t*-test (*P ≤ 0.05, ***P < 0.01, *n* = 10).

To evaluate the phenotypic response of soybean seedlings infested with SCN, plant height (Figure 3A) measurements were recorded at 0, 1, 3, 5, 7, 10, and 14 DAI. Throughout the 14-day period all mock infested plants remained green and healthy looking and the only clear visible symptoms of SCN infestation was stunting and slight chlorosis of the cotyledons and lower leaves in the later time points (Figure 3B). At days 0, 1, and 3 DAI no discernable difference was observed or measured between the infested and mock treated plants. At 5–7 DAI, infected plants tended to appear slightly shorter, however, there was no significant difference between the two treatments (Figure 3A). Significant (*t-*test (*P* ≤ 0.05) stunting was evident at 10 and 14 DAI. At 10 DAI SCN treated plants were over 1 cm (~9 %) shorter (11.22 ± 0.15 cm) compared to mock treated plants (12.26 ± 0.18 cm). At 14 DAI this height difference was over 1.9 cm (~16 %) with mock treated plants having an average height of 13.95 ± 0.12 cm, compared to infested plants with an average of 12.01 ± 0.13 cm.

### Identification of Common Plant Volatiles (CPVs)

For each of the 6 time points post infestation, we identified between 400 and 500 possible VOCs as indicated by integrated peak areas from the GC profile. However, following alignment as described in methods, many were not found to be reproducibly detected in most replicates for a given treatment i.e., had a low detection rate. Supplementary Table 2 shows the number of significant (permutation test *P* < 0.05) and non-significant volatile compounds identified between SCN and mock infested plants computed at the different detection rates. Although the number of non-significant volatiles were reduced with increasing detection percentages, the number of significant volatiles were not reduced until after the 50 % detection point. At the 50 % detection rate, the number of VOCs detected ranged from 149 (at 1 DAI) to 202 (5 DAI) per time point. 107 VOCs were found to be present at all time points (referred to as Common Plant VOCs, CPVs) and were used for further analysis (Table 1).

**Table 1:** Properties of the 107 Common Plant VOCs (CPVs).

					**1 DAI**			**3 DAI**			**5 DAI**			**7 DAI**			**10 DAI**			**14 DAI**		
**CPV**	**RT**	**Confirmed identity**	**Group**	**Pathway**	**Mock**	**SCN**	**F.C**.	**Mock**	**SCN**	**F.C**.	**Mock**	**SCN**	**F.C**.	**Mock**	**SCN**	**F.C**.	**Mock**	**SCN**	**F.C**.	**Mock**	**SCN**	**F.C**.
CPV41^*^	10.02	Undecane	1	Fatty Acid Derivative	1.09	1.06	0.97	0.70	0.81	1.16	0.81	0.97	1.20	1.13	1.48	1.31	0.89	1.18	1.33	1.19	1.46	1.24^*^
CPV33	8.78		1		0.70	0.64	0.93	0.49	0.57	1.17	0.50	0.58	1.16	0.71	0.92	1.29	0.66	0.82	1.25	0.79	0.88	1.11
CPV51^*^	11.88	Dodecane	1	Fatty Acid Derivative	1.03	1.05	1.02	0.80	0.99	1.24	0.93	1.08	1.17	1.05	1.55	1.48	0.82	1.17	1.42^*^	1.20	1.42	1.18
CPV60^*^	13.78		1		0.84	0.76	0.90	0.89	0.93	1.05	1.08	1.14	1.05	1.11	1.38	1.25	0.76	1.10	1.43^*^	1.38	1.64	1.19
CPV62	14.38		1		0.68	0.57	0.85	0.70	0.73	1.04	0.58	0.80	1.38	0.81	1.08	1.34	0.68	0.83	1.22	1.08	1.26	1.16
CPV66^*^	15.47		1		0.33	0.24	0.71	0.45	0.47	1.03	0.56	0.75	1.33	0.51	0.74	1.47	0.39	0.52	1.34	0.58	0.87	1.50^*^
CPV78	17.47		1		0.52	0.54	1.04	0.49	0.62	1.25	0.75	0.82	1.10	0.66	0.76	1.15	0.68	0.75	1.10	0.77	0.96	1.25
CPV7^*^	4.86	2,4-Dimethylheptane	1	Fatty Acid Derivative	2.58	2.59	1.01	2.27	2.82	1.24^*^	2.53	2.60	1.03	2.15	2.79	1.30^***^	1.76	2.06	1.17^*^	2.50	2.73	1.09
CPV13	6.19	Nonane	1	Fatty Acid Derivative	0.24	0.24	1.00	0.10	0.16	1.59	0.24	0.24	1.00	0.52	0.61	1.16	0.12	0.14	1.11	0.26	0.28	1.07
CPV35^*^	9.33	2,6-Dimethylheptane	1	Fatty Acid Derivative	0.96	0.89	0.93	0.74	0.84	1.13	0.87	0.87	1.00	0.79	1.04	1.32	0.68	0.95	1.40^**^	0.94	0.85	0.90
CPV34^*^	9.21	O-Decylhydroxylamine	1	Amino Acid Derivative	1.67	1.58	0.95	1.32	1.53	1.16	1.63	1.80	1.11	1.59	1.91	1.20	1.12	1.61	1.43^**^	1.64	1.56	0.95
CPV11^*^	6.06	Styrene	1	Phenylpropanoids and Benzenoids	0.58	0.59	1.01	0.41	0.57	1.37^*^	0.42	0.53	1.28^**^	0.43	0.62	1.43^***^	0.28	0.42	1.51^**^	0.46	0.43	0.94
CPV6^*^	4.76		1		0.30	0.21	0.69	0.08	0.11	1.33	0.27	0.29	1.07	0.15	0.23	1.52	0.13	0.20	1.57^*^	0.23	0.22	0.94
CPV49	11.33		1		0.41	0.43	1.04	0.33	0.45	1.37	0.58	0.58	0.99	0.62	0.84	1.35	0.37	0.55	1.46	0.69	0.74	1.07
CPV40^*^	9.94		1		0.47	0.41	0.88	0.49	0.61	1.25	0.22	0.18	0.84	0.33	0.50	1.54^*^	0.40	0.61	1.55	0.46	0.50	1.10
CPV37	9.58		1		0.34	0.36	1.08	0.28	0.32	1.14	0.21	0.15	0.69	0.35	0.51	1.44	0.31	0.41	1.35	0.44	0.42	0.96
CPV32^*^	8.71	D-Limonene	1	Terpenoid	0.86	0.90	1.05	1.48	1.55	1.05	0.88	1.61	1.83^*^	1.71	2.13	1.25	0.74	0.89	1.19	1.57	1.40	0.90
CPV21^*^	7.35		1		0.34	0.41	1.19	0.26	0.19	0.73	0.18	0.27	1.45	0.31	0.41	1.32	0.32	0.43	1.32^**^	0.43	0.38	0.90
CPV48^*^	11.26		1		0.91	1.06	1.17	0.77	0.81	1.06	0.88	1.19	1.35	0.96	1.13	1.18	0.71	1.28	1.81^*^	1.51	1.40	0.93
CPV70^*^	16.19		1		0.42	0.38	0.91	0.85	1.17	1.38	1.20	1.43	1.19	0.55	0.83	1.50^*^	0.46	0.82	1.77^*^	0.87	1.25	1.43
CPV43^*^	10.35		1		0.62	0.53	0.86	0.47	0.53	1.14	0.36	0.45	1.25	0.46	0.79	1.73	0.30	0.63	2.10^*^	0.59	0.79	1.34
CPV63^*^	14.92		1		0.23	0.16	0.67	0.20	0.24	1.16	0.13	0.19	1.50	0.28	0.46	1.62^*^	0.21	0.45	2.16^*^	0.46	0.62	1.35
CPV73^*^	16.49		1		22.36	23.37	1.05	19.47	21.66	1.11	25.07	32.40	1.29	23.04	35.75	1.55^*^	16.83	25.96	1.54^**^	29.35	43.69	1.49^**^
CPV67^*^	15.65		1		0.43	0.45	1.05	0.46	0.49	1.07	0.71	0.78	1.10	0.53	0.93	1.76^*^	0.48	0.77	1.60^**^	0.80	1.04	1.30^*^
CPV76^*^	17.16	Butylated Hydroxytoluene	1	Phenylpropanoids and Benzenoids	7.72	8.90	1.15	8.34	10.37	1.24	11.70	16.94	1.45	7.42	13.73	1.85^***^	10.54	19.26	1.83^***^	13.61	21.67	1.59^***^
CPV74^*^	16.69	2,6-Di-T-butyl-4-methylene-2,5-cyclohexadiene-1-one	1	Phenylpropanoids and Benzenoids	1.36	1.71	1.26	0.93	1.67	1.79	1.25	1.91	1.53^**^	0.86	1.97	2.30^***^	0.81	1.61	1.98^***^	1.29	1.84	1.43^*^
CPV58	13.55		1		0.12	0.23	1.94	0.24	0.24	0.97	0.08	0.16	2.08	0.20	0.22	1.07	0.16	0.18	1.15	0.27	0.36	1.34
CPV103	28.02		1		0.75	1.18	1.57	2.19	2.65	1.21	1.54	2.21	1.43	1.42	1.36	0.96	1.03	1.15	1.11	0.77	1.78	2.32
CPV71	16.31		1		0.14	0.23	1.66	0.08	0.17	2.06	0.34	0.43	1.26	0.27	0.34	1.28	0.18	0.18	1.03	0.25	0.36	1.41
CPV65^*^	15.30	Tetradecane	1	Fatty Acid Derivative	0.81	0.94	1.17	0.60	0.97	1.62^***^	0.87	1.21	1.40^**^	0.73	1.01	1.38	0.61	0.81	1.32^**^	0.83	1.03	1.24^*^
CPV57	13.49		1		0.27	0.28	1.03	0.20	0.28	1.39	0.31	0.26	0.85	0.15	0.34	2.24	0.19	0.24	1.24	0.55	0.53	0.97
CPV2^*^	3.43		1		0.46	0.48	1.05	0.45	0.45	1.00	0.70	0.53	0.76^**^	0.20	0.46	2.31^*^	0.40	0.47	1.18	0.36	0.52	1.44
CPV53	12.13		1		0.31	0.53	1.71	0.30	0.36	1.20	0.33	0.17	0.51	0.27	0.50	1.83	0.21	0.40	1.92	0.43	0.52	1.20
CPV54^*^	13.00		1		0.31	0.31	1.05	0.30	0.31	1.04	0.37	0.30	0.80	0.33	0.43	1.30	0.29	0.54	1.89^**^	0.53	0.77	1.44^*^
CPV39^*^	9.83		1		0.88	0.89	1.01	0.49	0.57	1.16	0.78	0.72	0.92	0.71	1.13	1.58	0.53	1.17	2.20^**^	0.95	1.00	1.05
CPV30^*^	8.57		1		0.39	0.34	0.88	0.21	0.15	0.72	0.23	0.17	0.76	0.23	0.37	1.60	0.19	0.34	1.77^*^	0.27	0.32	1.20
CPV44	10.49		1		0.32	0.27	0.84	0.19	0.21	1.08	0.34	0.18	0.53	0.29	0.37	1.26	0.11	0.27	2.44	0.27	0.29	1.09
CPV1^*^	3.32		1		2.18	2.00	0.92	2.23	2.01	0.90	2.72	1.97	0.72^**^	0.08	0.79	9.40^**^	1.31	1.77	1.35	2.48	1.82	0.74^*^
CPV45^*^	10.61		1		0.17	0.24	1.40	0.13	0.17	1.29	0.14	0.09	0.67	0.16	0.17	1.02	0.04	0.25	6.10^**^	0.20	0.31	1.57
CPV31^*^	8.66	2-Ethylhexanol	1	Fatty Acid Derivative	0.97	1.21	1.25	0.27	0.52	1.90	0.37	0.10	0.26^*^	0.20	0.20	1.00	0.23	0.76	3.38^*^	0.22	0.34	1.54
CPV55^*^	13.16		1		0.12	0.20	1.59	0.09	0.05	0.53	0.17	0.26	1.51	0.19	0.24	1.27	0.04	0.19	4.68^*^	0.23	0.16	0.69
CPV20^*^	7.23		1		0.12	0.41	3.33^**^	0.14	0.41	2.90^*^	0.06	0.25	4.35	0.26	0.71	2.73^**^	0.18	0.48	2.64^**^	0.28	0.18	0.65
CPV104^*^	29.66		1		1.30	12.45	9.57^**^	1.46	1.82	1.25	2.58	3.42	1.33	2.83	2.63	0.93	2.58	3.51	1.36	2.37	2.27	0.96
CPV19^*^	7.17	Camphene	2	Terpenoid	1.23	0.98	0.80	0.91	0.74	0.81	0.26	0.25	0.94	0.36	0.52	1.43	0.33	0.14	0.42	1.22	0.76	0.62^*^
CPV15^*^	6.47		2		0.28	0.15	0.52	0.17	0.19	1.10	0.11	0.14	1.31	0.11	0.10	0.92	0.27	0.13	0.47^**^	0.17	0.17	0.96
CPV87^*^	20.01	Ethylhexyl benzoate	2	Phenylpropanoids and Benzenoids	1.08	0.52	0.48^***^	0.81	0.52	0.63^**^	0.98	0.66	0.67^*^	0.91	0.52	0.57^**^	0.93	0.53	0.57^***^	0.69	0.62	0.89
CPV4^*^	4.10	Toluene	2	Phenylpropanoids and Benzenoids	2.19	1.78	0.81	0.55	0.24	0.44^*^	1.63	1.44	0.88	1.60	1.26	0.78	1.13	0.85	0.75^*^	1.87	1.79	0.96
CPV23	7.53		2		0.22	0.21	0.93	0.09	0.04	0.40	0.22	0.19	0.87	0.19	0.21	1.11	0.07	0.09	1.30	0.18	0.14	0.77
CPV69^*^	15.97		2		0.33	0.35	1.08	0.40	0.23	0.57^*^	0.65	0.38	0.59^*^	0.37	0.22	0.59	0.33	0.32	0.97	0.36	0.23	0.63^*^
CPV96^*^	24.57		2		0.33	0.06	0.19	1.34	0.63	0.47	2.60	2.02	0.78	1.82	0.61	0.33^**^	1.21	1.05	0.87	2.77	1.61	0.58
CPV98	25.72	Heptacosane	3	Fatty Acid Derivative	0.26	0.20	0.79	0.36	0.33	0.93	0.45	0.44	0.98	0.54	0.41	0.77	0.64	0.58	0.91	0.24	0.24	1.02
CPV64	15.17		3		0.66	0.54	0.82	0.72	0.68	0.94	0.75	0.67	0.89	0.63	0.46	0.74	0.74	0.63	0.85	0.70	0.75	1.07
CPV99	25.86		3		0.38	0.34	0.89	0.57	0.56	0.98	0.57	0.39	0.68	0.84	0.64	0.76	0.95	0.85	0.90	0.63	0.69	1.08
CPV59	13.64		3		1.83	1.47	0.80	1.54	1.29	0.84	2.43	2.08	0.86	2.17	1.88	0.87	1.42	1.57	1.11	1.95	1.93	0.99
CPV14	6.23		3		0.55	0.39	0.71	0.62	0.69	1.10	0.51	0.43	0.86	0.39	0.32	0.80	0.61	0.65	1.07	0.67	0.57	0.85
CPV82	18.36		3		1.64	1.06	0.64	1.07	1.10	1.03	1.39	1.47	1.06	1.04	0.66	0.64	0.70	0.91	1.29	0.85	0.76	0.89
CPV46	10.73		3		0.12	0.12	0.94	0.08	0.06	0.72	0.16	0.16	0.97	0.33	0.16	0.49	0.11	0.17	1.48	0.16	0.18	1.15
CPV107	30.52		3		3.47	3.10	0.89	7.18	4.88	0.68	10.28	8.03	0.78	9.19	7.14	0.78	5.00	9.01	1.80	9.44	10.77	1.14
CPV81	18.25		3		0.21	0.15	0.71	0.13	0.13	0.96	0.31	0.31	1.02	0.22	0.20	0.91	0.18	0.21	1.18	0.17	0.19	1.10
CPV50^*^	11.72	1-Dodecene	3	Fatty Acid Derivative	1.30	1.01	0.78	0.98	0.93	0.95	1.15	1.34	1.17	1.20	1.25	1.04	1.00	1.31	1.31^**^	1.21	1.33	1.10
CPV52	12.00	Decanal	3	Fatty Acid Derivative	5.02	3.67	0.73	5.13	5.05	0.98	5.83	5.83	1.00	6.84	7.19	1.05	5.58	5.80	1.04	7.29	7.06	0.97
CPV42	10.12	Nonanal	3	Fatty Acid Derivative	5.46	4.32	0.79	5.67	6.08	1.07	4.60	4.68	1.02	4.99	5.75	1.15	4.80	5.31	1.11	5.61	5.60	1.00
CPV28	8.17		3		1.46	1.22	0.84	1.32	1.27	0.96	1.36	1.27	0.93	1.52	1.75	1.15	1.29	1.72	1.33	1.67	1.67	0.99
CPV25	7.86	6-methyl-5-hepten-2-one	3	Terpenoid	5.31	3.73	0.70	5.21	4.47	0.86	5.79	5.65	0.98	4.72	5.64	1.19	4.21	6.55	1.56	5.68	5.91	1.04
CPV27	8.09	Decane	3	Fatty Acid Derivative	0.51	0.51	1.01	0.31	0.29	0.94	0.42	0.42	1.01	0.61	0.70	1.15	0.34	0.44	1.31	0.40	0.36	0.91
CPV22	7.40	1,2,4-Trimethylbenzene	3	Phenylpropanoids and Benzenoids	0.68	0.69	1.01	0.26	0.24	0.95	0.38	0.46	1.20	0.53	0.57	1.09	0.23	0.29	1.26	0.62	0.57	0.92
CPV24^*^	7.69	(-)-β-Pinene	3	Terpenoid	2.36	2.19	0.93	1.99	1.87	0.94	1.70	1.71	1.01	2.01	1.79	0.89	0.73	1.09	1.49^*^	1.85	1.86	1.01
CPV9	5.55	3-Ethylhexane	3	Fatty Acid Derivative	1.46	1.44	0.99	1.05	1.14	1.09	1.45	1.31	0.91	1.25	1.47	1.18	1.14	1.09	0.96	1.47	1.35	0.92
CPV12	6.11	o-Xylene	3	Phenylpropanoids and Benzenoids	0.88	0.80	0.91	0.28	0.31	1.09	0.60	0.63	1.05	0.45	0.57	1.26	0.39	0.40	1.03	0.58	0.61	1.05
CPV10	5.68	p-Xylene	3	Phenylpropanoids and Benzenoids	1.79	1.70	0.95	0.41	0.51	1.25	1.15	1.17	1.02	0.93	0.90	0.97	0.71	0.63	0.88	1.15	1.09	0.95
CPV56	13.28	(E)-2-hexenal	3	Fatty Acid Derivative	2.10	1.87	0.89	1.72	1.51	0.88	2.36	2.46	1.04	2.00	1.93	0.96	2.30	2.42	1.06	4.94	4.57	0.93
CPV17	6.85	α-Pinene	3	Terpenoid	7.38	6.92	0.94	5.00	4.79	0.96	4.17	4.31	1.03	4.58	4.38	0.96	1.61	1.63	1.01	4.16	4.16	1.00
CPV36	9.46		3		0.57	0.50	0.88	0.47	0.47	1.00	0.57	0.67	1.18	0.55	0.62	1.12	0.40	0.35	0.89	0.62	0.56	0.90
CPV93	23.24		3		0.34	0.23	0.66	0.39	0.45	1.14	0.58	0.59	1.02	0.55	0.49	0.89	0.66	0.61	0.93	0.19	0.24	1.30
CPV86^*^	19.94		3		0.43	0.33	0.77	0.55	0.53	0.97	0.53	0.58	1.10	0.54	0.44	0.83	0.68	0.56	0.81^*^	0.42	0.53	1.28
CPV94	23.91		3		0.35	0.25	0.72	0.37	0.36	1.00	0.35	0.46	1.32	0.34	0.23	0.66	0.24	0.24	1.00	0.32	0.39	1.22
CPV18	7.07		3		0.29	0.23	0.78	0.28	0.22	0.78	0.32	0.33	1.05	0.27	0.29	1.07	0.31	0.30	0.97	0.27	0.32	1.18
CPV101^*^	27.88		3		0.95	0.71	0.75^*^	1.40	1.19	0.85	1.69	1.88	1.11	1.80	1.56	0.86	2.17	2.01	0.92	1.69	1.80	1.07
CPV97	25.25		3		0.21	0.12	0.60	0.45	0.32	0.72	0.48	0.56	1.16	0.51	0.57	1.12	0.69	0.64	0.92	0.26	0.33	1.28
CPV83	19.41		3		0.20	0.18	0.89	0.12	0.12	1.03	0.27	0.31	1.12	0.31	0.28	0.91	0.20	0.24	1.18	0.39	0.56	1.43
CPV80	17.79	Pentadecane	3	Fatty Acid Derivative	0.15	0.14	0.97	0.17	0.16	0.98	0.25	0.31	1.24	0.20	0.17	0.86	0.25	0.26	1.05	0.20	0.26	1.25
CPV100	27.54	Docosane	3	Fatty Acid Derivative	0.17	0.14	0.82	0.33	0.28	0.85	0.41	0.46	1.12	0.47	0.43	0.91	0.52	0.55	1.06	0.15	0.21	1.34
CPV5^*^	4.71		3		1.96	1.96	1.00	1.72	1.66	0.96	1.90	1.88	0.99	1.70	1.72	1.02	1.85	1.74	0.94	1.70	1.97	1.16^***^
CPV47	10.83		3		0.49	0.47	0.96	0.30	0.26	0.87	0.49	0.46	0.93	0.47	0.43	0.90	0.15	0.16	1.09	0.54	0.66	1.22
CPV26^*^	8.03		3		1.51	1.49	0.99	0.80	0.89	1.11	0.86	0.91	1.06	0.89	1.04	1.17	0.76	0.81	1.06	0.90	1.43	1.59^*^
CPV89	20.79		3		0.18	0.20	1.09	0.28	0.22	0.81	0.24	0.37	1.51	0.54	0.53	0.97	0.49	0.32	0.65	0.39	0.45	1.16
CPV16	6.68		3		0.22	0.19	0.83	0.14	0.14	1.01	0.27	0.23	0.84	0.13	0.19	1.40	0.11	0.07	0.62	0.26	0.34	1.33
CPV88^*^	20.53	Heptadecane	3	Fatty Acid Derivative	0.38	0.22	0.57	0.41	0.30	0.75	0.50	0.55	1.09	0.63	0.45	0.72	0.81	0.50	0.61^***^	0.28	0.54	1.96^*^
CPV106^*^	29.93		3		0.37	0.32	0.85	0.66	0.79	1.19	0.51	0.65	1.27	0.54	0.39	0.71	0.71	0.48	0.68^*^	0.19	0.43	2.33
CPV77	17.36		4		0.42	0.48	1.14	0.42	0.41	0.97	0.72	0.58	0.81	0.48	0.31	0.64	0.41	0.42	1.01	0.33	0.25	0.76
CPV105^*^	29.79		4		3.15	5.41	1.72^**^	4.96	4.64	0.93	6.27	6.23	0.99	6.17	5.20	0.84	7.68	7.09	0.92	6.86	7.12	1.04
CPV75	16.84		4		0.79	1.28	1.62	0.32	0.36	1.11	1.14	1.02	0.90	0.70	0.68	0.97	0.62	0.92	1.50	0.77	1.07	1.39
CPV29	8.24	(Z)-3-Hexen-1-ol acetate	4	Fatty Acid Derivative	0.21	0.40	1.92	0.96	1.40	1.47	1.00	0.66	0.66	1.64	1.34	0.82	0.57	0.39	0.69	0.78	0.53	0.68
CPV85^*^	19.78		4		0.22	0.28	1.28	0.27	0.38	1.40	0.52	0.51	0.97	0.42	0.24	0.56^*^	0.36	0.35	0.96	0.27	0.31	1.15
CPV79	17.66		4		0.18	0.17	0.95	0.16	0.25	1.57	0.25	0.23	0.92	0.28	0.15	0.54	0.27	0.20	0.75	0.16	0.23	1.45
CPV95	24.52		4		0.64	0.61	0.96	0.46	0.91	1.96	0.88	1.66	1.88	1.45	1.09	0.75	1.42	1.06	0.74	0.57	0.69	1.21
CPV68	15.81		4		0.09	0.11	1.16	0.02	0.05	2.38	0.23	0.14	0.62	0.10	0.08	0.81	0.04	0.07	1.83	0.20	0.15	0.74
CPV3^*^	3.81	2-Butanone	4	Fatty Acid Derivative	0.29	0.28	0.98	0.21	0.61	2.92^*^	0.25	0.29	1.16	0.16	0.09	0.58	0.10	0.17	1.67	0.50	0.67	1.34^**^
CPV91	22.08		4		0.05	0.07	1.43	0.36	0.39	1.07	0.18	0.15	0.85	0.56	0.31	0.55	0.60	0.24	0.40	0.34	0.91	2.64
CPV90^*^	21.11		4		0.11	0.23	2.18	0.20	0.18	0.86	0.29	0.34	1.17	0.40	0.19	0.47^*^	0.29	0.19	0.66	0.11	0.35	3.17^*^
CPV72	16.45		5		0.23	0.06	0.24	0.11	0.24	2.09	0.40	0.28	0.70	0.32	0.29	0.90	0.28	0.33	1.20	0.24	0.17	0.71
CPV38^*^	9.64		5		0.39	0.14	0.36^*^	0.09	0.18	2.15	0.19	0.12	0.63	0.31	0.23	0.75	0.10	0.14	1.39	0.17	0.17	1.01
CPV84	19.46		5		0.14	0.06	0.43	0.09	0.13	1.56	0.23	0.15	0.66	0.22	0.26	1.21	0.17	0.20	1.23	0.11	0.22	2.07
CPV102	27.96		5		0.07	0.03	0.43	0.08	0.23	2.77	0.14	0.23	1.71	0.17	0.21	1.21	0.40	0.46	1.16	0.21	0.14	0.67
CPV92	22.73		5		0.45	0.18	0.40	0.72	0.58	0.80	0.83	1.07	1.28	0.94	0.87	0.93	1.11	0.92	0.83	0.36	0.55	1.53
CPV8	4.92		5		0.10	0.04	0.43	0.10	0.07	0.71	0.09	0.14	1.51	0.12	0.13	1.13	0.16	0.16	0.95	0.17	0.22	1.31
CPV61	14.25		5		0.11	0.02	0.21	0.18	0.16	0.88	0.19	0.26	1.37	0.22	0.30	1.35	0.15	0.22	1.45	0.29	0.32	1.13

* *indicates statistically significant Infested/Mock ratio (permutation test P < 0.05; n = 20). Red shading indicates significant CPV emission for SCN infestation. Blue shading indicates significant suppression in SCN infested plant*.

*Data shows mock and SCN concentrations (nmol/L Octane) along with log^2^ fold change (SCN/Mock) for all 6 time points, confirmed identification with metabolic pathway and GC retention time. CPVs grouped in order of hierarchical clusters (1–5)*.

** *P < 0.001*,

*** *P < 0.0001*.

### Analysis of Common Plant Volatiles (CPVs)

#### Overall Temporal Expression of CPVs

**Figure 4:**
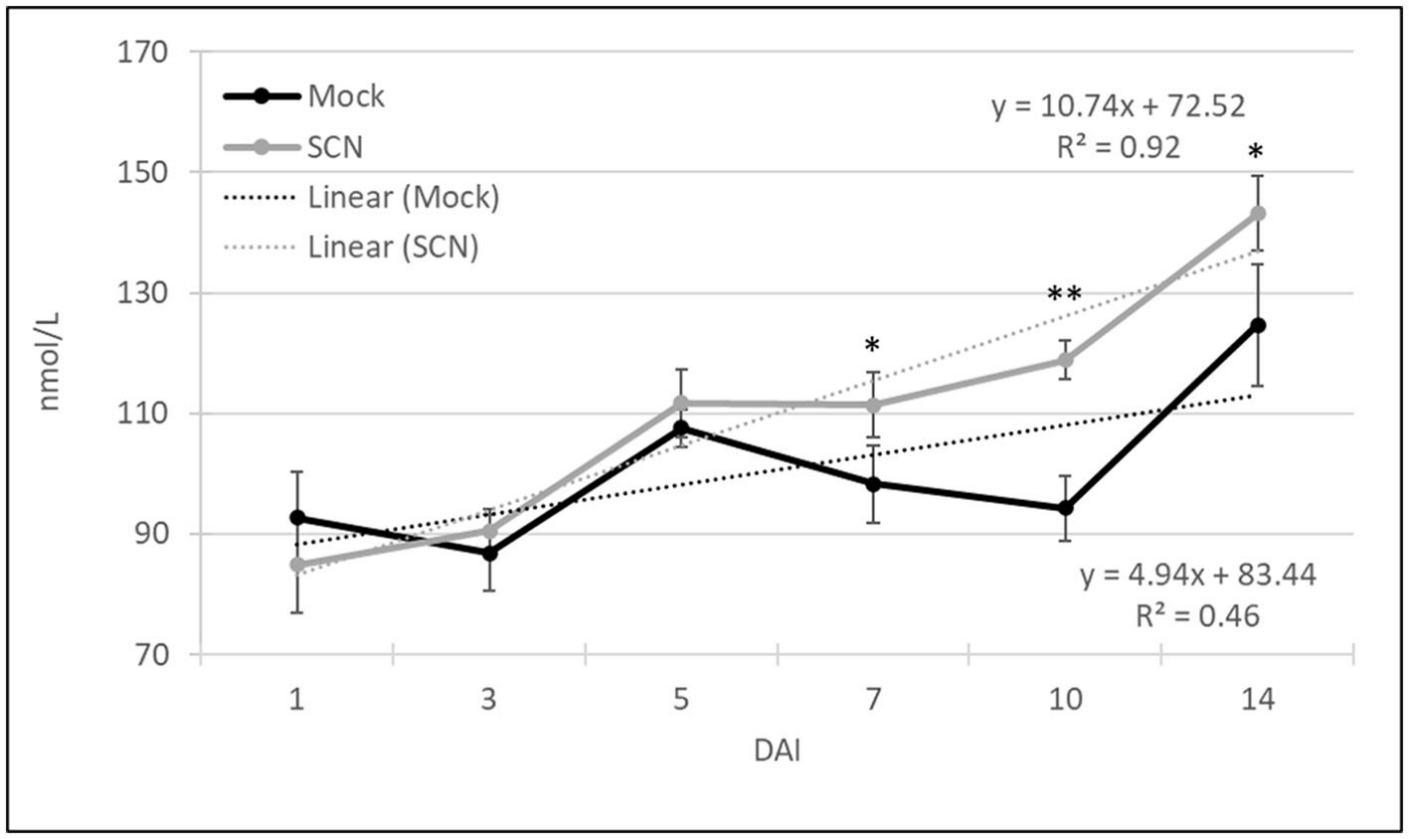
Total volatile emission from all 107 CPVs over time for SCN and mock treated plants. Graph includes linear regression slopes for both mock and SCN infested plants and goodness of fit (R^2^). The data indicates average ± SE total VOC concentration and significant differences based on Student's t-test (*P ≤ 0.05, **P < 0.01, *n* = 10).

To investigate the overall production of VOCs over time and in response to SCN infestation, we summed the VOCs (normalized to octane units) of all 107 CPVs for each time point (Figure 4). After one DAI both the SCN infested and mock plants yielded similar total volatiles. Over the ensuing 2-week period both untreated and SCN infested plants showed increased volatile production, likely in part due to the increase in plant material (Figure 3). Notably, total VOCs were significantly increased (>20 %) at 7–14 DAI in SCN compared to mock treated plants. Linear regression showed a more than two-fold increased slope for SCN (y = 10.74x+72.52 with an *R*^2^ = 0.92) compared to mock treatment (*y* = 4.94x + 83.44 with an *R*^2^ = 0.46). In sum, these data show SCN infestation results in enhanced VOC production.

#### Identified CPV Abundance and Metabolic Pathways

From the 107 CPVs, we were able to confirm the identity of 33 different VOCs. Each of these 33 CPVs was categorized into one of four metabolic pathways: terpene, phenylpropanoid and benzenoid, fatty acid, and amino acid derivatives (Table 1). To evaluate the relative contribution to the overall VOC profile, the average abundance for each of the 33 CPVs across all 6 time points for each treatment was calculated. Overall, the 33 identified CPVs for the SCN treatment had a sum of 62.98 nmol/L averaged across all 6 time points. This was made up of 42 % fatty acid derivatives, 34 % phenylpropanoids and benzenoids, 21 % terpenes, and 3 % amino acid derivatives. The mock treatment had a total of 56.20 nmol/L averaged across the 6 time points comprising 45 % fatty acid derivatives, 28 % phenylpropanoids and benzenoids, 24 % terpenes, and 3 % amino acid derivatives. For both treatments, fatty acid derivatives had the highest abundance with Decanal (CPV52) being the most abundantly produced VOC, followed by Nonanal (CPV42) and (E)-2-hexenal (CPV56). Within the terpenes, 6-methyl-5-hepten-2-one (CPV25) was the most abundant terpene for both treatments. Other highly abundant terpenes included α-Pinene (CPV17) and β-Pinene (CPV24). Butylated Hydroxytoluene (CPV76) was the most abundant CPV from the phenylpropanoids and benzenoids pathway for both treatments. Toluene (CPV4) and 2,6-Di-T-butyl-4-methylene-2,5-cyclohexadiene-1-one (CPV74) were other abundant phenylpropanoids and benzenoids. The least abundant pathway for both treatments was the amino acid derivatives. Only one CPV was identified in this pathway, O-Decyl Hydroxylamine (CPV34).

### Hierarchical Clustering Analysis of CPV Expression Patterns in Response to SCN Infestation

**Figure 5:**
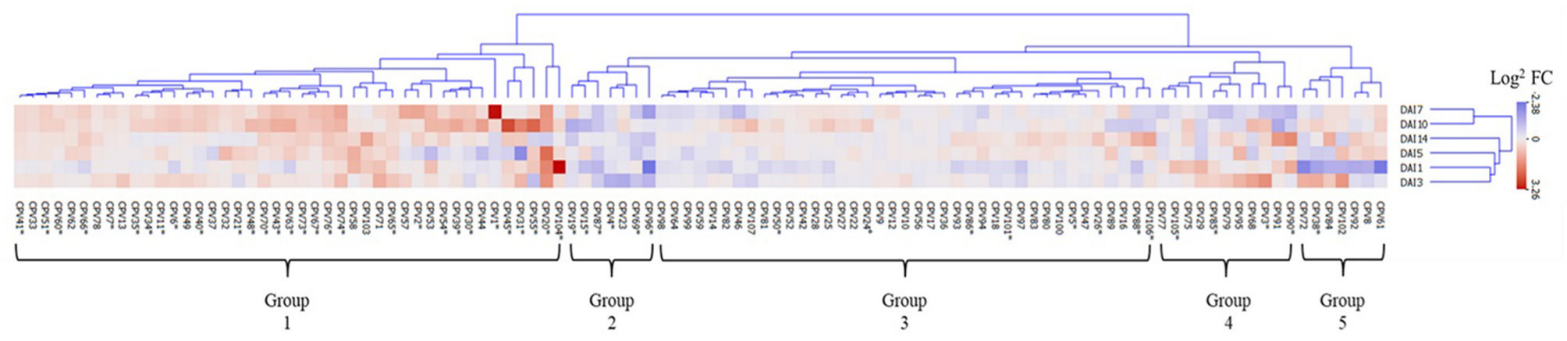
Clustering analysis of 107 CPVs in response to SCN infestation. The heat map was arranged according to CPV log^2^ fold change (Infestation/Mock). Five groups were identified, arranged from left to right as elevated, suppressed, no change, and oscillating expression Infested/mock ratios, respectively. Red, blue, and white indicate elevated, suppressed, and no change in expression ratios of volatile emissions. The six rows illustrate the expression patterns of CPVs during the time points 1, 3, 5, 7, 10, and 14 DAI. * indicates statistically significant Infested/Mock ratio (permutation test *P* < 0.05).

To further dissect the temporal relationships of the emitted VOCs, we conducted hierarchical clustering analysis of the log^2^ fold change of SCN infestation verse mock treatments. The 107 CPVs clustered into 5 distinct groups with neighboring time points generally being more closely clustered (Figure 5). Group 1 revealed CPVs that had greater expression in SCN infested plants compared to mock plants. Out of the 43 CPVs in this group, 31 were significantly up expressed (designated by ^*^) at one or more time points. Of these 31, 11 had confirmed identification; 2,4-Dimethylheptane (CPV7), Styrene (CPV11), 2-Ethylhexanol (CPV31). D-Limonene (CPV32), O-Decyl Hydroxylamine (CPV34), 2,6-Dimethylheptane (CPV35), Undecane (CPV41), Dodecane (CPV51), Tetradecane (CPV65), 2,6-Di-T-butyl-4-methylene-2,5-cyclohexadiene-1-one (CPV74), and Butylated Hydroxytoluene (CPV76). These were categorized as being derived from different metabolic pathways; 6 fatty acid derivatives, 1 terpenes, 1 amino acid derivative, along with 3 volatiles belonging to the phenylpropanoid and benzenoid pathway (Table 1). Group 2 contained 7 CPVs with 6 being significant and suppressed in SCN infested plants. Of the 6, we confirmed the identification of Toluene (CPV4), Camphene (CPV19), and Ethylhexyl benzoate (CPV87). This group contained 1 terpene and 2 volatiles from the phenylpropanoid and benzenoid pathway (Table 1). CPVs with moderate or no discernible changes in expression levels were clustered into group 3. Nevertheless, 8 of the 39 CPVs in this group were significant with only 3 having confirmed identities. These were 1-Dodecene (CPV50), β-Pinene (CPV24), and Heptadecane (CPV88). Significant CPVs in this group contained 1 fatty acid derivative, 1 terpene, and 1 phenylpropanoid and benzenoid volatiles (Table 1). Both groups 4 and 5 showed fluctuating expression patterns, but were differentiated by suppression in SCN treatment at day 7 and day 1, respectively. Group 4 contained 11 CPVs with 4 significant and group 5 had 7 CPVs with 1 significant. One significant CPV, 2-Butanone (CPV3), was confirmed in these 2 groups which belonged to the fatty acid derivative pathway (Table 1). In sum, hierarchical clustering revealed a large group of VOCs being elevated (group 1) and a small group (group 2) being suppressed due to SCN infestation. Eleven compounds were identified in the former group and 3 in the latter.

### Principal Component Analysis of Significant and Identified CPVs Over 6 Time Points

**Figure 6:**
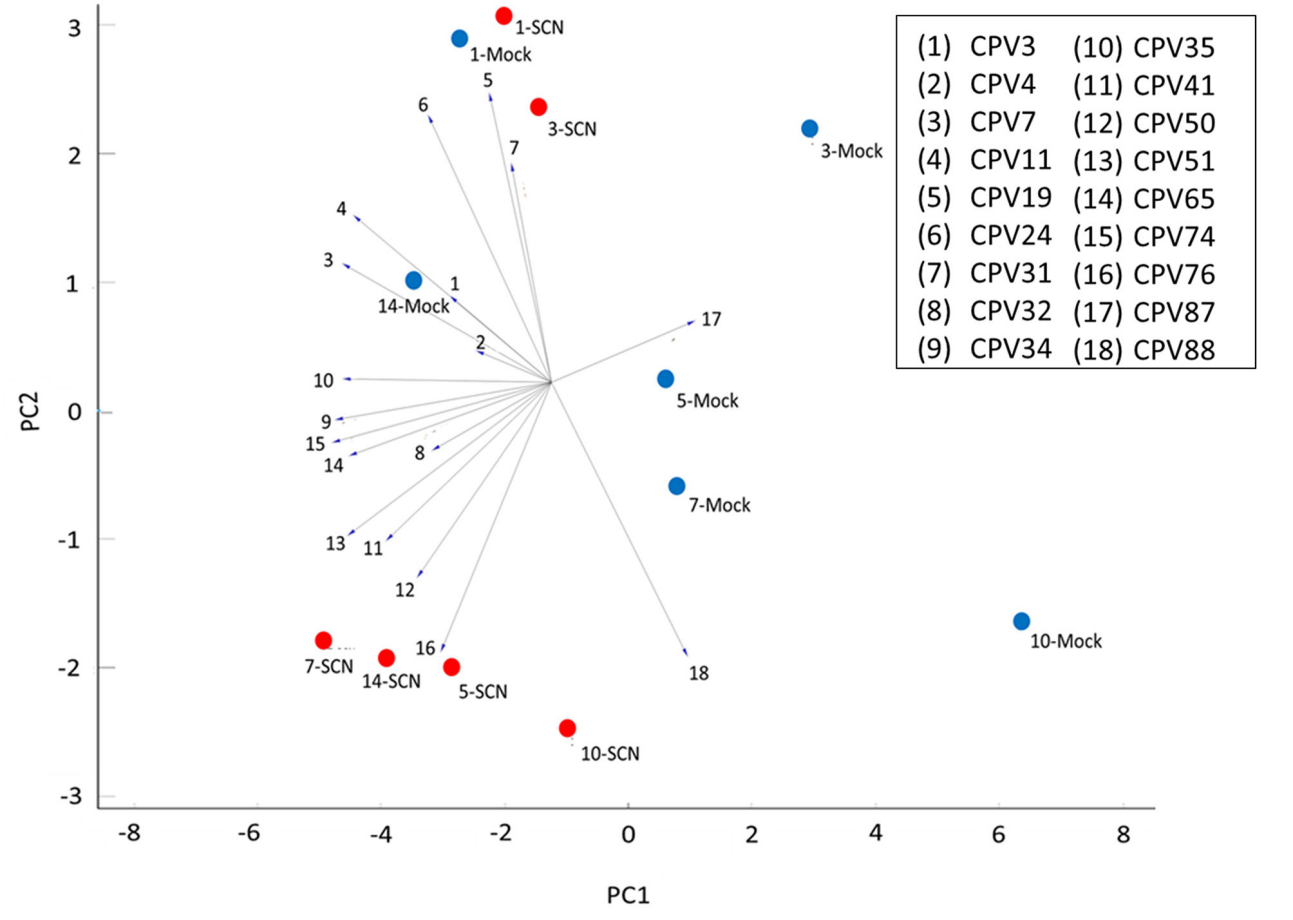
Linear projection of principal component analysis (PCA) of identified and significant CPVs for all 6 time points. Explained variance was 60% with a cumulative variance of 0.602 and component variance of 0.195. PCA was based on 18 CPVs over 1, 3, 5, 7, 10, and 14 DAI. Mock time points are assigned blue circles and SCN time points are assigned red circles.

We used Principal Component Analysis (PCA) coupled with a linear projection to explore relationships between the CPVs and time points of both SCN and mock treated soybean plants (Figure 6). SCN treatment at 1 and 3 DAI clustered closely with their mock time points, however, SCN 5, 7, 10, and 14 DAI were notably separated from their mock treated controls. Of the 4 the main volatiles responsible for the separation, 3 were the fatty acid derivatives Undecane (CPV41), 1-Dodecene (CPV50), and Dodecane (CPV51), and the other being a phenylpropanoid, Butylated Hydroxytoluene (CPV76). All of which were found in Cluster group 1, except for CPV50, and all were significantly enhanced in SCN infested plants (Table 1). Five other significant members of Cluster group 1 (Figure 5) including D-Limonene (CPV32), O-Decyl Hydroxylamine (CPV34), 2,6-Dimethylheptane (CPV35), Tetradecane (CPV65), and 2,6-Di-T-butyl-4-methylene-2,5-cyclohexadiene-1-one (CPV74) were important for separating the 5–14 DAI SCN time points from the other treatments. Thus, overall, 8 of the 11 VOCs identified were found to be consistently associated with SCN infestation by hierarchical clustering and PCA. One other notable CPV, Ethylhexyl benzoate (CPV87), a member of cluster group 2 (Figure 5), was shown to be suppressed during infestation, and was negatively correlated with the SCN 5–14 DAI, and positively correlated with the majority of the mock time points.

### Characterization of Foliar CPVs Associated With Early SCN Infestation

**Figure 7:**
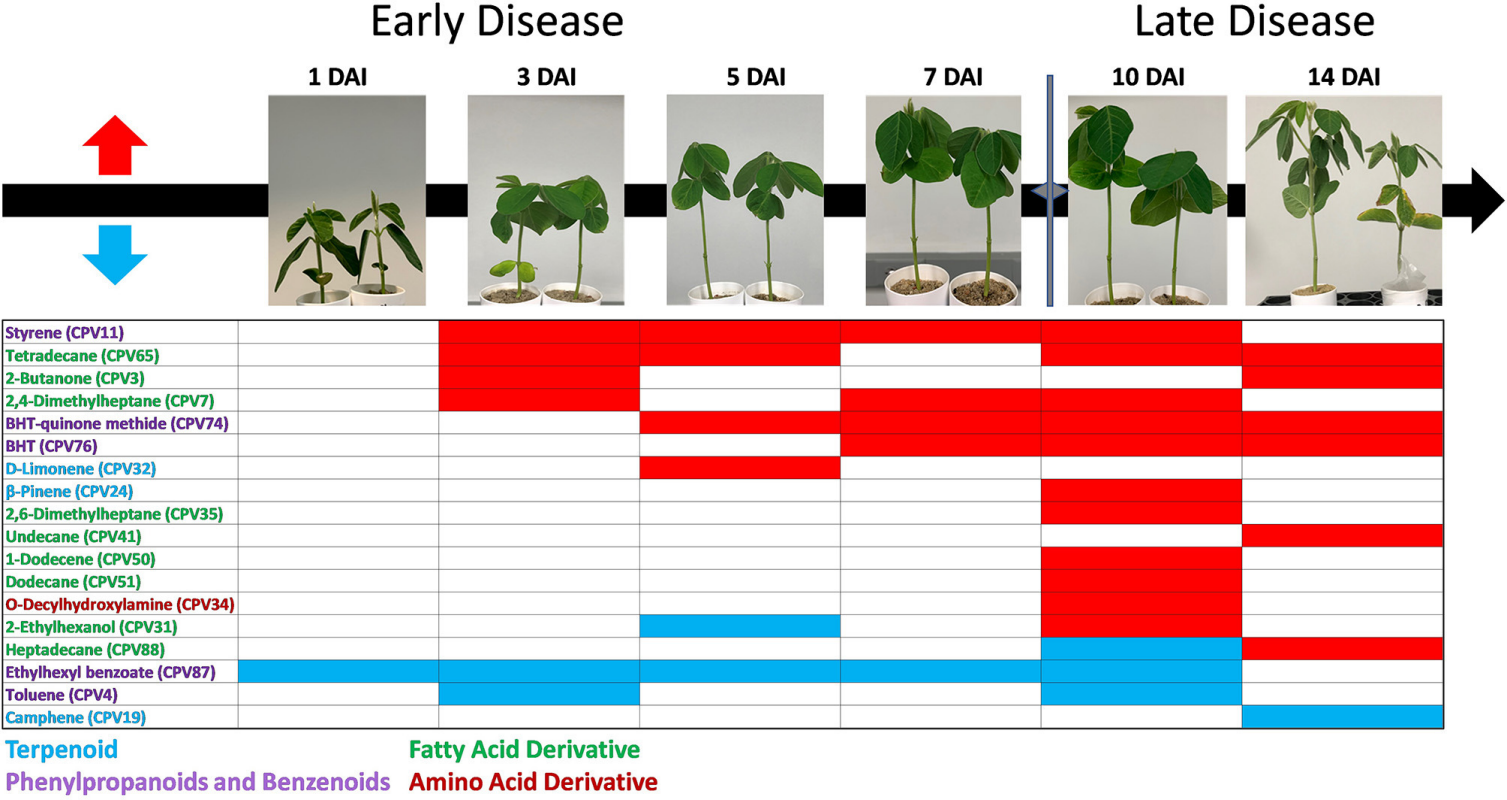
Temporal expression patterns of identified CPVs with significant differences in expression between SCN infested and mock treatments. Time points were 1, 3, 5, 7, 10, and 14 DAI. Red blocking indicates statistically significant emission and blue blocking indicates significant suppression of VOCs by infested plants (permutation test *P* < 0.05; *n* = 20). BHT-quinone methide and BHT abbreviations for 2,6-Di-T-butyl-4-methylene-2,5-cyclohexadiene-1-one and Butylated Hydroxytoluene, respectively. Images show representative mock (left) and SCN infested (right) plants at different timepoints.

Since stunting symptoms first became significant at 10 DAI (Figure 1), CPVs of interest for the early detection of SCN would be evident at time points 1–7 DAI. Out of 107 CPVs, 18 were found to have confirmed identities and be significant at 1 or more time points. Of these 18 CPVs, 10 were found to be significant at the early time points with 7 being enhanced and 3 suppressed by SCN infestation (Figure 7). 2-Butanone (CPV3) and 2,4-Dimethylheptane (CPV7) were significantly elevated at 3 DAI along with Styrene (CPV11) and Tetradecane (CPV65), which were also significant at 5 DAI. Both D-Limonene (CPV32) and 2,6-Di-T-butyl-4-methylene-2,5-cyclohexadiene-1-one (CPV74) were also significantly elevated at 5 DAI. At 7 DAI, 2,4, Dimethylheptane (CPV7), Styrene (CPV11), 2,6-Di-T-butyl-4-methylene-2,5-cyclohexadiene-1-one (CPV74), and Butylated Hydroxytoluene (CPV76) were significantly induced. Notably, 4 of these CPVs (D-Limonene CPV32, Tetradecane CPV65, 2,6-Di-T-butyl-4-methylene-2,5-cyclohexadiene-1-one CPV74 and Butylated Hydroxytoluene CPV76) were also identified by hierarchical clustering and PCA analysis as being discerning of SCN infestation. Of the 3 CPVs that exhibited reduced expression, only Ethylhexyl benzoate (CPV87) was significantly suppressed at all early time points, while Toluene (CPV4) was only suppressed at 3 DAI and 2-Ethylhexanol (CPV31) was suppressed at 5 DAI. In summary, D-Limonene CPV32, Tetradecane CPV65, 2,6-Di-T-butyl-4-methylene-2,5-cyclohexadiene-1-one CPV74 and Butylated Hydroxytoluene CPV76 were consistently found to be elevated prior to SCN symptom development and Ethylhexyl benzoate CPV87 to be consistently suppressed.

## Discussion

Our work reveals that infestation of soybean roots with SCN results in the elevated release of VOCs from foliar tissues and that this rise occurs prior to symptom appearance (Figures 3, 4). Above ground responses including defense activation, changes in hormone and metabolite levels have been observed following root damage, but examples of emission of foliar VOCs have been reported in few instances (Bezemer and Dam, 2005). One such example is the herbivory of *Brassica nigra* (black mustard) roots by *Delia radicum* which affects the behavior of *Cotesia glomerata*, a parasitoid of the leaf herbivore *Pieris brassicae* through alteration of the VOC bouquet. Given the choice, *C. glomerata* prefers to oviposit in hosts feeding on plants without root damage. Volatile analysis showed that plants with root damage emitted high levels of sulfur volatiles, which are highly toxic to insects. When compared to undamaged root plants, infested plants had lower levels of volatiles reported to be attractants for carnivorous and herbivorous insects, such as beta-farnesene (Soler et al., 2007). Another study demonstrated the *Aphis glycines* preferred non-infested soybean plants compared to SCN infested plants when given the choice, suggestive of a role for VOCs (Hong et al., 2010). To date there is little knowledge on plant-nematode associated VOCs. Though the volatile itself was not studied in the defense of soybeans to SCN, it has been shown that the overexpression of the (E, E)- α-farnesene terpene synthase gene of soybean plays a role in nematode defense (Lin et al., 2017). In other work, VOCs recovered from soybean extracts including specific alcohols, ketones, furans, and predominantly aldehydes volatiles were shown to inhibit the growth of *Aspergillus flavus* and production of aflatoxin B1 (Cleveland et al., 2009).

A primary hypothesis motivating this work was to evaluate whether foliar VOCs were elevated prior to symptom development because they likely would represent key biomarkers for early disease detection. During the course of infestation, in addition to SCN resulting in an overall increase in total volatile emissions, individual VOCs from particular metabolic pathways differed as well. Infested plants had higher overall levels of terpene, fatty acid and in particular phenylpropanoids and benzenoids derivatives compared to mock treated plants. Inspection of Hierarchical clustering analysis and Linear Projection PCA revealed 4 VOCs, D-Limonene (CPV32), Tetradecane (CPV65), 2,6-Di-T-butyl-4-methylene-2,5-cyclohexadiene-1-one (CPV74), and Butylated Hydroxytoluene (CPV76) were all significantly elevated at the early stages of infestation (Figures 5–7). Though not initially included in the VOCs of interest, Styrene (CPV11) was also seen to have elevated emissions in the earlier time points and most likely was not grouped with the others in the PCA due to the combination of pooled mid to late time points. Like the other volatiles of interest, Styrene (CPV11) is a member of cluster group 1. It should also be noted that Ethylhexyl benzoate (CPV87) was strongly suppressed during infestation at both early and late time points.

Limonene is a common monoterpene with D-Limonene (CPV32) being the most abundant isomer found as a major component of citrus oils in fruit peel and in small concentrations in other fruits and vegetables (Mosandl et al., 1990). Limonene has anti-pest properties including nematicidal activity (Oka et al., 2012). Essential oils extracted from *L. juneliana* and *L. turbinate*, which contained 23.1 % and 43.3–60.6 % limonene content, respectively, was able to kill more than 80 % of the juveniles of the root-knot nematode *Meloidogyne sp*. (Duschatzky et al., 2004). Limonene has also been shown to protect tomato plants from whitefly infestation when dispensed in pure volatile form or emitted from a companion plant, such as marigolds, grown near them (Conboy et al., 2019). The fatty acid derivative, Tetradecane (CPV65) has been shown to exhibit toxicity to *M. incognita* eggs and juveniles under laboratory conditions (Ansari et al., 2020). It has also been shown to be emitted by melons infested with whiteflies as well as serving to attract parasitoids for plant defense (Silveira et al., 2018). Styrene (CPV11), a phenylpropanoid and benzenoid derivative, an important monomer for commercial products such as plastics, synthetic rubbers and paints, does occur naturally (Miller et al., 1994). Styrene and derivatives have been found in plants, including soybean and as a by-product of fungal and microbial metabolism (Shirai and Hisatsuka, 1979; Arpaia et al., 2011; Kim et al., 2020). Styrene produced by *Bacillus mycoides* in the rhizosphere of tomato plants exhibited high nematocidal activity against *M. incognita* (Luo et al., 2018). Butylated Hydroxytoluene (CPV76), a phenylpropanoid and benzanoid derivative, is a natural antioxidant, and has been found in plant oils (Yehye et al., 2015). It has been shown to alter the behavior of western corn rootworm (*Diabrotica virgifera*) larvae, a major pest of maize, by attracting healthy larvae to nematode infested (*Heterorhabditis bacteriophora)* cadavers and continuing the predation process (Zhang et al., 2019). While not showing a direct association with nematode infestation of plants, it does indicate that Butylated Hydroxytoluene (CPV76) is utilized by nematodes to increase reproductive success and could be a volatile linked to nematode infestation. Its role as an antioxidant, may suggest a role in countering plant defense through ameliorating consequences of oxidative stress (Babu and Wu, 2008). Along with styrene and Butylated Hydroxytoluene (CPV76), 2,6-Di-T-butyl-4-methylene-2,5-cyclohexadiene-1-one (CPV74) (also known as BHT-quinone methide) is a phenylpropanoid and benzenoid derivative. It is an oxidized form of BHT found in plants, however, there is little information on its function in the literature. Analogs of this compound have been identified in bacteria, fungi, plants and animals, which have been shown to exhibit potent toxicity against all tested organisms (Zhao et al., 2020). The phenylpropanoid and benzenoid derivative, Ethylhexyl benzoate (CPV87), which is suppressed in soybean infestation in this study, has been detected in several plant species, but very little is known about its biological function (Musayeib et al., 2016; Bajer et al., 2018).

Our findings also provide additional insight into the spectrum of VOCs produced by soybean plants, with fatty acid derivatives, phenylpropanoids and benzenoids, and terpenes being the most abundant metabolic sources. Several of the volatiles found in our study correlate with previous volatile studies of soybean plants (Kim et al., 2020). In our studies, the most abundant class of VOCs were fatty acid derivatives. These are derived from the C18 unsaturated fatty acids, linoleic and linolenic acids. The fatty acids are shunted into the lipoxygenase (LOX) pathway, where they are oxygenated and further metabolized (Feussner and Wasternack, 2002). Volatiles generated through the fatty acid pathway include, hexanal, (Z)-3-hexenol, nonanal, and methyl jasmonate, which have all been indicated in plant response to stress (Dickens et al., 1992; Dudareva et al., 2013; Zhang et al., 2017; Hammerbacher et al., 2019). In our studies, the most abundant were Decanal (CPV52), Nonanal (CPV42), and (E)-2-hexenal (CPV56), consecutively. Both Nonanal (CPV42) and (E)-2-hexenal (CPV56) have been identified from soybeans, as well as have been shown to be potent antifungal volatiles against *Aspergillus flavus* (Cleveland et al., 2009; Kim et al., 2020). Decanal (CPV52) and to a lesser extent Nonanal (CPV42), produced by insect damaged potato tubers, have been shown to serve as powerful attractants to entomopathogenic nematodes (Laznik and Trdan, 2016).

Phenylpropanoids and benzenoids were the second most abundant class of VOCs produced by SCN infestation. These VOCs are derived from aromatic amino acid phenylalanine (Phe) synthesized through chorismate, the product of the shikimate pathway (Knudsen et al., 2006; Dudareva et al., 2013). Compounds associated with this group have been shown to be involved in plant growth, light response, and are key mediators of organismal interactions (Biała and Jasiński, 2018). In addition to Butylated Hydroxytoluene (CPV76) and its oxidized derivative 2,6-Di-T-butyl-4-methylene-2,5-cyclohexadiene-1-one (CPV74) mentioned above, Toluene (CPV4) was abundantly detected from soybeans (Kim et al., 2020). Although a common petroleum-derived product, Toluene was originally discovered in pine oil. Interestingly, Toluene has been shown to be spontaneously emitted by both sunflowers and pine in response to both biotic and abiotic stress (Heiden et al., 1999; Isidorov et al., 2003).

Terpene derivatives were the third most abundant group of VOCs emitted by soybean. Of the different pathways, the terpene pathway comprises the largest and most structurally diverse group (McGarvey and Croteau, 1995). Terpenes are produced through the mevalonate pathway and the methyl D- erythritol 4-phosphate (MEP) pathway with over 20,000 terpenes have been found in animals, plants, bacteria, fungi, and archaea (Hunter et al., 2003). Major plant terpenes of interest include limonene, α-pinene, β-pinene, linalool, and β-caryophyllene, which have all been shown to be implicated in a plant response to stress (Fantaye et al., 2015; Ercioglu et al., 2018; Shi et al., 2019; Wang et al., 2019; Silva et al., 2020). In addition to D-limonene mentioned above, we found 6-methyl-5-hepten-2-one (CPV25), α-Pinene (CPV17), and β-Pinene (CPV24) to be the most abundant derivatives detected. 6-methyl-5-hepten-2-one, also known as sulcatone, has a fruity, citrusy odor. It is an oily VOC emitted by many plants, particularly in oils of citronella, lemon-grass and palmarosa and is a powerful mosquito pheromone (Logan et al., 2010; Dekel et al., 2019). α-Pinene (CPV17), and β-Pinene (CPV24) are isomers of pinene and are the main compounds released by forest trees and have been detected to be produced by soybeans (Kim et al., 2020). Both volatiles have a woody and pine scent and are most often generated together in plants (Geron et al., 2000).

Though not well studied in plants, the final pathway of interest for this study are VOCs derived from amino acid derivatives that often yield floral scents and fruit aromas (Dudareva et al., 2013). From this pathway various aldehydes, alcohols, esters, acids, and nitrogen- and sulfur containing compounds are formed that play vital roles in plant defense *via* attracting natural predators of the attacking herbivore insect (Dudareva et al., 2006; War et al., 2011). VOCs are often derived from alanine, valine, leucine, isoleucine, and methionine (Knudsen et al., 2006). We only identified one amino acid derivative in soybean, O-Decyl Hydroxylamine (CPV34), an N-containing compound, which has not been well studied, but has been shown to be expressed in certain rice grains (Ocan et al., 2020).

Our findings that SCN infestation results in elevated release of particular foliar VOCs, some prior to symptom appearance, may provide opportunity for the development of new diagnostic tools. It remains to be determined whether significant levels of VOCs are released in an incompatible resistant interaction and/or whether other soilborne stresses trigger similar or different VOC profiles (Lin et al., 2017). SCN infestations are difficult to detect and usually take the form of stunting instead of the more obvious symptoms such as leaf spots, galls, and cankers (Niblack et al., 2006). Disease diagnostics based on volatile analysis is reliable, but due to the state-of-the-art equipment currently being GC-MS or variants thereof, it is typically time consuming, labor-intensive and expensive that cannot measure volatiles in real-time and can only be done in a laboratory setting (Sankaran et al., 2010; Madufor et al., 2018). The electronic nose (e-nose) system represents a technology for disease detection that could be deployed in the field. The technology typically employs an array of sensors that can be programmed to respond to different volatile blends and through pattern recognition can identify specific biotic stresses such as plant diseases (Pardo and Sberveglieri, 2005; Loutfi et al., 2015; Fitzgerald et al., 2017). A number of hand-held sensors are commercially available; however, additional refinements are still needed for their general utility in field settings.

In conclusion, our results show that root infestation of young soybean plants with the noxious pest SCN results in the elevated release of VOCs from foliar tissues before visible symptoms are readily apparent. Moreover, we found that a number of VOCs, notably Styrene (CPV11), D-Limonene (CPV32), Tetradecane (CPV65), 2,6-Di-T-butyl-4-methylene-2,5-cyclohexadiene-1-one (CPV74) and Butylated Hydroxytoluene (CPV76) were consistently elevated and Ethylhexyl benzoate (CPV87) to be consistently suppressed. These VOCs likely represent valuable biomarkers for the early detection of SCN infestation. Additional studies are needed to confirm whether these biomarkers are detectable under field settings.

## Data Availability Statement

The original contributions presented in the study are included in the article/Supplementary Material, further inquiries can be directed to the corresponding authors.

## Author Contributions

NC, YO, EŞ, ÖO, and RD contributed to conceptualization, design of the experiment, methodology, and fund acquisition. MW contributed tissue propagation and infestation. NC performed the experiments and contributed to data curation and manuscript preparation. EŞ and RD revised the manuscript. BA fabricated software. YO contributed to supervision, data analysis, and editing. All authors contributed to the article and approved the submitted version.

## Funding

Funding for this research was provided by BASF Plant Science under award number 2018-3163 and 2019-1403 Task Order JA000618 to RD, YO, and ÖO. Additional funding and resources for this project were supplied by the Tri-Institutional Molecular Mycology and Pathogenesis Training Program, Duke University/National Institutes of Health 3021730 (2021-0302, 2020-0808, 2018-3424) to NC, and NC State University College of Agriculture and Life Sciences Research Foundation to RD and ÖO.

## Conflict of Interest

MW was employed by BASF Plant Science. The remaining authors declare that the research was conducted in the absence of any commercial or financial relationships that could be construed as a potential conflict of interest. The authors declare that this study received funding from BASF Plant Science. The funder had the following involvement in the study: MW contributed to plant propagation and infestation of plants with nematodes.

## Publisher's Note

All claims expressed in this article are solely those of the authors and do not necessarily represent those of their affiliated organizations, or those of the publisher, the editors and the reviewers. Any product that may be evaluated in this article, or claim that may be made by its manufacturer, is not guaranteed or endorsed by the publisher.
