# Supplementary figures and images for: Soybean Cyst Nematodes Influence Aboveground Plant Volatile Signals Prior to Symptom Development
Source: Front Plant Sci. 2021 Sep 29;12:749014. doi: 10.3389/fpls.2021.749014 (PMC8513716; doi:10.3389/fpls.2021.749014)

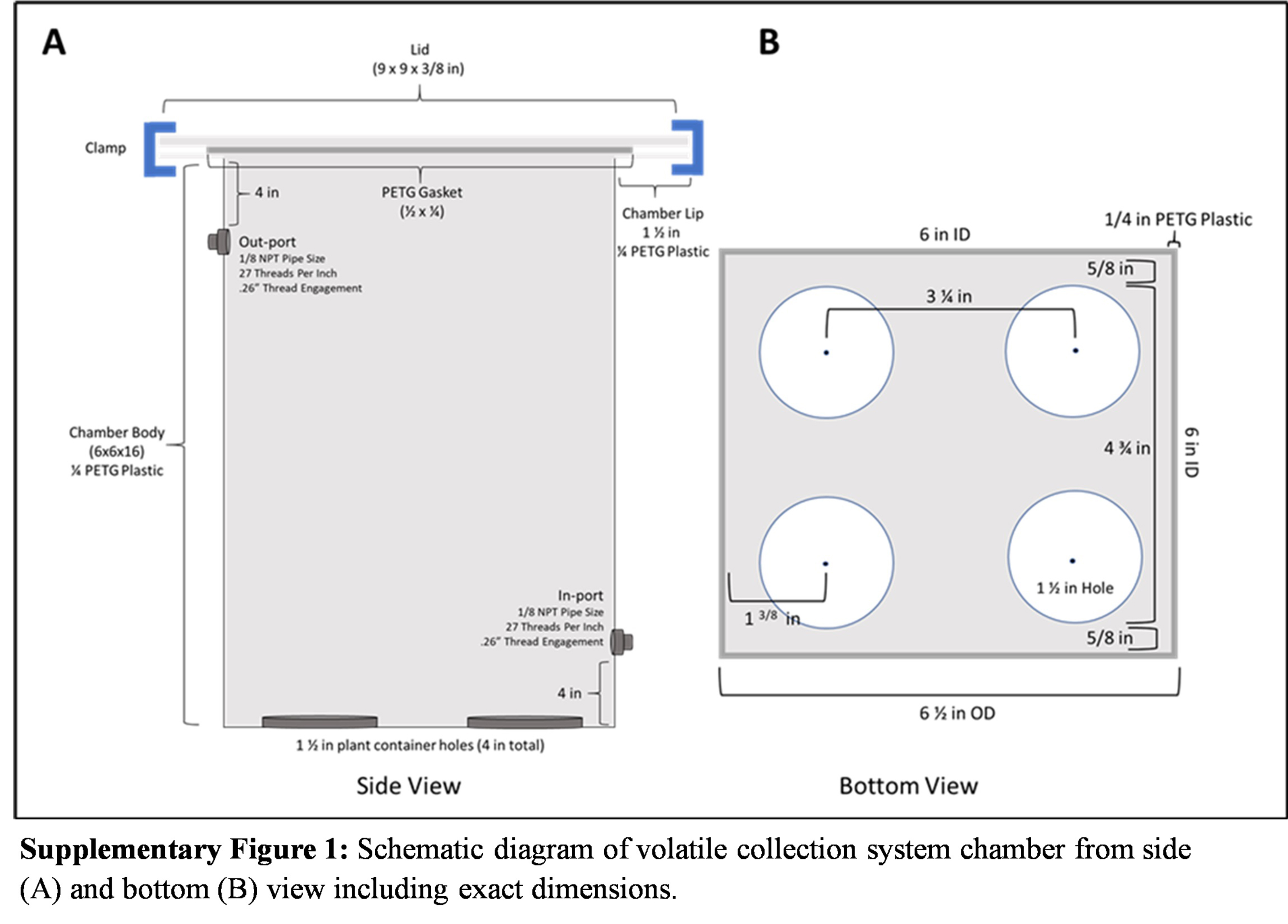

Supplement: Supplementary file 3 [file Image_1.JPG]
